# Quantification of Local Electric Field Changes at the Active Site of Cytochrome *c* Oxidase by Fourier Transform Infrared Spectroelectrochemical Titrations

**DOI:** 10.3389/fchem.2021.669452

**Published:** 2021-04-27

**Authors:** Federico Baserga, Jovan Dragelj, Jacek Kozuch, Hendrik Mohrmann, Ernst-Walter Knapp, Sven T. Stripp, Joachim Heberle

**Affiliations:** ^1^Department of Physics, Experimental Molecular Biophysics, Freie Universität Berlin, Berlin, Germany; ^2^Macromolecular Modelling Group, Institute of Chemistry and Biochemistry, Freie Universität Berlin, Berlin, Germany; ^3^Modeling of Biomolecular Systems, Technische Universität Berlin, Berlin, Germany

**Keywords:** vibrational Stark effect, carbon monoxide, proton transfer, electrostatic potential, redox chemistry, electron transfer, infrared spectroscopy

## Abstract

Cytochrome *c* oxidase (C*c*O) is a transmembrane protein complex that reduces molecular oxygen to water while translocating protons across the mitochondrial membrane. Changes in the redox states of its cofactors trigger both O_2_ reduction and vectorial proton transfer, which includes a proton-loading site, yet unidentified. In this work, we exploited carbon monoxide (CO) as a vibrational Stark effect (VSE) probe at the binuclear center of C*c*O from *Rhodobacter sphaeroides*. The CO stretching frequency was monitored as a function of the electrical potential, using Fourier transform infrared (FTIR) absorption spectroelectrochemistry. We observed three different redox states (R_4_CO, R_2_CO, and O), determined their midpoint potential, and compared the resulting electric field to electrostatic calculations. A change in the local electric field strength of +2.9 MV/cm was derived, which was induced by the redox transition from R_4_CO to R_2_CO. We performed potential jump experiments to accumulate the R_2_CO and R_4_CO species and studied the FTIR difference spectra in the protein fingerprint region. The comparison of the experimental and computational results reveals that the key glutamic acid residue E286 is protonated in the observed states, and that its hydrogen-bonding environment is disturbed upon the redox transition of heme a_3_. Our experiments also suggest propionate A of heme a_3_ changing its protonation state in concert with the redox state of a second cofactor, heme a. This supports the role of propionic acid side chains as part of the proton-loading site.

## Introduction

The eukaryotic respiratory chain exploits electron-rich substrates to pump protons from the mitochondrial matrix into the intermembrane space; the resulting proton gradient provides energy for adenosine triphosphate (ATP) production (Mitchell, 1966). The terminal oxidase of the mitochondrial respiratory chain receives electrons from cytochrome *c* and is referred to as “complex IV,” or cytochrome *c* oxidase (C*c*O) (Wikstrom, 1977). While we know that C*c*O catalyzes the reduction of molecular oxygen (O_2_) to water, the molecular mechanism by which the enzyme couples O_2_ reduction and proton translocation are not entirely understood.

Cytochrome *c* oxidase is a transmembrane protein complex naturally found in many organisms (García-Horsman et al., 1994). Bacterial C*c*Os are typically less complex than their eukaryotic relatives, which simplifies the biotechnological production. The terminal oxidase from *Rhodobacter sphaeroides* (*Rs*C*c*O) is often used as a model organism for eukaryotic isoenzymes, since its active site shares high-sequence identity to mammalian oxidases, e.g., from *Bos taurus* (*Bt*C*c*O) (Hosler et al., 1992; García-Horsman et al., 1994). *Rs*C*c*O comprises four subunits: subunit I is fundamental to the function of the enzyme as it harbors the two cofactors heme a and heme a_3_. The latter forms the catalytic binuclear center (BNC), together with a copper ion (Cu_B_) that is coordinated by three histidine residues (Pereira et al., 2001).

**Figure 1:**
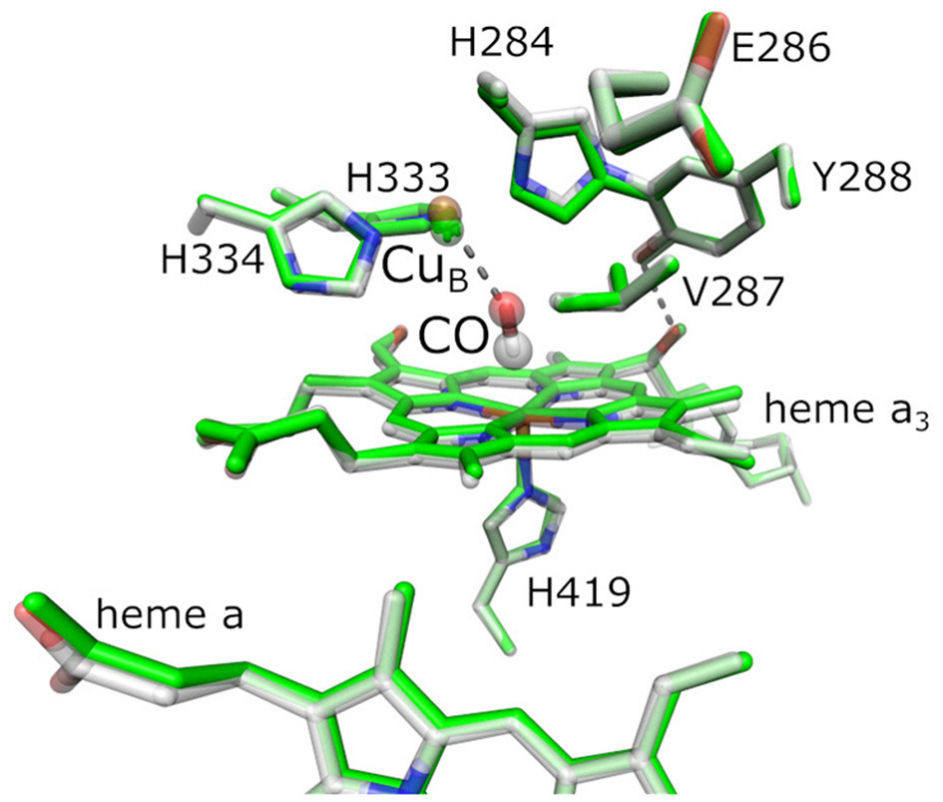
Active site of bovine cytochrome *c* oxidase (*Bt*C*c*O) in the oxidized state (2DYR, in green) and in the fully reduced, CO-inhibited state (3AG1, with colored atoms). The binuclear center (BNC) comprehends heme a_3_, the copper ion Cu_B_, and a number of conserved residues as labeled. The highlighted residues H334, H333, H284, E286, Y288, V287, and H419 are labeled according to the *Rs*C*c*O nomenclature and are analogous to the actual residues H291, H290, H240, E242, Y244, V241, and H376 in *Bt*C*c*O shown here.

Reduction of O_2_ is catalyzed at the BNC, where O_2_ binds to the central iron of heme a_3_ and Cu_B_. Other ligands are able to coordinate heme a_3_ or Cu_B_ as well, in particular carbon monoxide (CO), nitric oxide, cyanide, and isocyanate (Jain and Kassner, 1984; Brzezinski and Malmström, 1985; Mitchell and Rich, 1994; Brunori, 2001). Figure 1 shows the BNC of *Bt*C*c*O in the oxidized form, overlaid by the fully reduced CO-inhibited form.

The catalytic cycle of O_2_ reduction comprises several redox intermediates. In the reductive phase, cytochrome *c* provides electrons through direct electron transfer to the dinuclear copper site (Cu_A_), which is located close to the surface of C*c*O. Cu_A_ represents the start of a “conductive wire” leading to heme a, heme a_3_, and, finally, to Cu_B_ (Hill, 1993; Sezer et al., 2015). The number of electrons at the four cofactors of C*c*O determines the three redox states, which are stable after reducing the enzyme. Starting from the oxidized state (O) (Michel et al., 1998), the two-electron-reduced state (R_2_) is formed in which Cu_A_ and heme a are oxidized, while heme a_3_ and Cu_B_ are both reduced (Greenwood et al., 1974; Brzezinski and Malmström, 1985). At strongly negative electric potential all four cofactors are reduced (FR or R_4_) (Hellwig et al., 1998; Brzezinski and Gennis, 2008). The O state dominates in the presence of O_2_; whereas the R_2_ and R_4_ states can be stabilized through chemical reduction (Namslauer et al., 2002; Schäfer et al., 2018). The R_4_ and O states have been analyzed *via* x-ray diffraction crystallography (Yoshikawa et al., 1998), electron paramagnetic resonance (EPR) spectroscopy (Sharpe et al., 2008), resonance Raman (RR) spectroscopy (Woodruff et al., 1981), and Fourier transform infrared (FTIR) spectroscopy (Heitbrink et al., 2002). The intermediates of the physiologically relevant R_2_ state are more difficult to trap using chemical methods, and demand transient methods like UV/Vis flow-flash spectroscopy (Gibson and Greenwood, 1963; Brzezinski and Malmström, 1985; Brzezinski and Gennis, 2008; Schäfer et al., 2018). However, UV/Vis spectroscopy is insensitive to protonation states of amino acid side chains, which are critical to understanding proton transfer, hydrogen-bonding changes, and other mechanistic details of C*c*O catalysis.

Cytochrome *c* oxidase pumps protons across the mitochondrial membrane against an electrochemical gradient. The protons necessary for the catalytic reaction are delivered *via* conserved amino acid trajectories referred to as “D” and “K” pathways, with the “chemical protons” for O_2_ reduction being provided exclusively through the latter (Ädelroth and Gennis RB, 1998). In the K pathway, the proton uptake correlates with the initial reduction of Cu_A_ and heme a. The proton translocation against the transmembrane proton gradient is driven by the so-called proton-loading site (PLS), which couples with the redox-reaction occurring at the BNC (Belevich et al., 2007). It has been proposed that the PLS is located next to the propionate groups of either heme a or heme a_3_ (Kaila et al., 2011; Sezer et al., 2017). In the D pathway, a glutamate residue controls the proton flux (E286 in *Rs*C*c*O, cf. Figure 1) that also plays a key role in proton translocation (Ädelroth et al., 1997; Nyquist et al., 2003). The carboxylic side chain of E286 was shown to be protonated in the O, R_2_, and R_4_ states of the redox cycle (Nyquist et al., 2003). A possible proton transfer scenario from E286 to the PLS may involve the reduction of heme a and the BNC (Belevich et al., 2006). Belevich et al. (2007) showed a correlation between membrane electrostatic potential and redox changes upon electron injection and suggested that redox changes at heme a, as well as pKa differences, are key factors.

Fourier transform infrared difference spectroscopy allows addressing changes in protonation states and hydrogen-bonding patterns (Lorenz-Fonfria, 2020), but transient techniques are difficult to apply due to the lack of specific triggers (Schleeger et al., 2009). Alternatively, *operando* FTIR spectroscopy, in combination with electrochemical titrations, can facilitate the detection of elusive species in order to assign vibrational bands to different redox states, specific amino acid side chains, or redox sites (Hellwig et al., 1998; Gorbikova et al., 2006; Dodia et al., 2013). Introducing an internal probe like CO as an axial ligand to heme a_3_ provides complementary feedback of functional relevance. As is clear from X-ray crystallography (Figure 1), the presence of CO does not introduce significant structural changes at the BNC when compared to the oxidized protein, rendering CO a molecular reporter and O_2_ surrogate (Yoshikawa et al., 1998). Due to its high frequency and a large dipole moment, the C≡O stretching vibration is easily detected by IR spectroscopy. Furthermore, the CO ligand exhibits a high Stark-tuning rate, enabling to report on the changes in the local electrostatic field through an electrochromic shift (vibrational Stark effect, VSE) (Park et al., 1999; Boxer, 2009).

In this work, we use *operando* FTIR difference spectroelectrochemistry in attenuated total reflection (ATR) configuration (Nyquist et al., 2004; Senger et al., 2017) to investigate CO-inhibited *Rs*C*c*O. This approach facilitates CO gas binding and redox titrations from the fully reduced to the oxidized state. We determined the midpoint potential, acquired the difference spectra, and discussed the changes in the hydrogen-bonding pattern and protonation for each of the identified redox states. The measured VSE of the CO ligand is compared to electrostatic computations, calculating electric field changes at the BNC for different redox and protonation states.

## Materials and Methods

### Sample Preparation

Wild-type C*c*O from *R. sphaeroides* was expressed, purified, and stored in phosphate buffer [10 mM phosphate, pH 8, with 0.1% n-dodecyl-β-D-maltosid (DDM)] at −80°C. Ahead of the experiment, the samples were inserted into liposomes to simulate the physiological environment of the enzyme (Robinson and Capaldi, 1977). We prepared two 1 mg/ml solutions of dipalmitoylphosphatidylcholine (DPPC) vesicles, as well as isotopically carbon-substituted ^13^C_40_-DPPC (Cambridge Isotope Laboratories) vesicles in 50 mM phosphate buffer (pH 8) with 100 mM NaCl. The vesicles were extruded through a filter with pore size of 100 nm. After washing the DDM-solubilized C*c*O with the same phosphate buffer, we reconstituted the sample in the lipid vesicles by detergent removal upon addition of 30 mg of biobeads over a period of 12 h. The molar ratio of lipids per C*c*O molecule was ~200:1.

The resulting samples were washed four times with the final buffer and pelleted by centrifugation, yielding two different samples: DPPC-reconstituted C*c*O and ^13^C_40_-DPPC-reconstituted C*c*O in H_2_O buffer (50 mM phosphate, pH 8, with 100 mM NaCl). The samples in D_2_O were prepared by *in situ* buffer exchange directly on the ATR crystal.

### Electrochemistry

**Figure 2:**
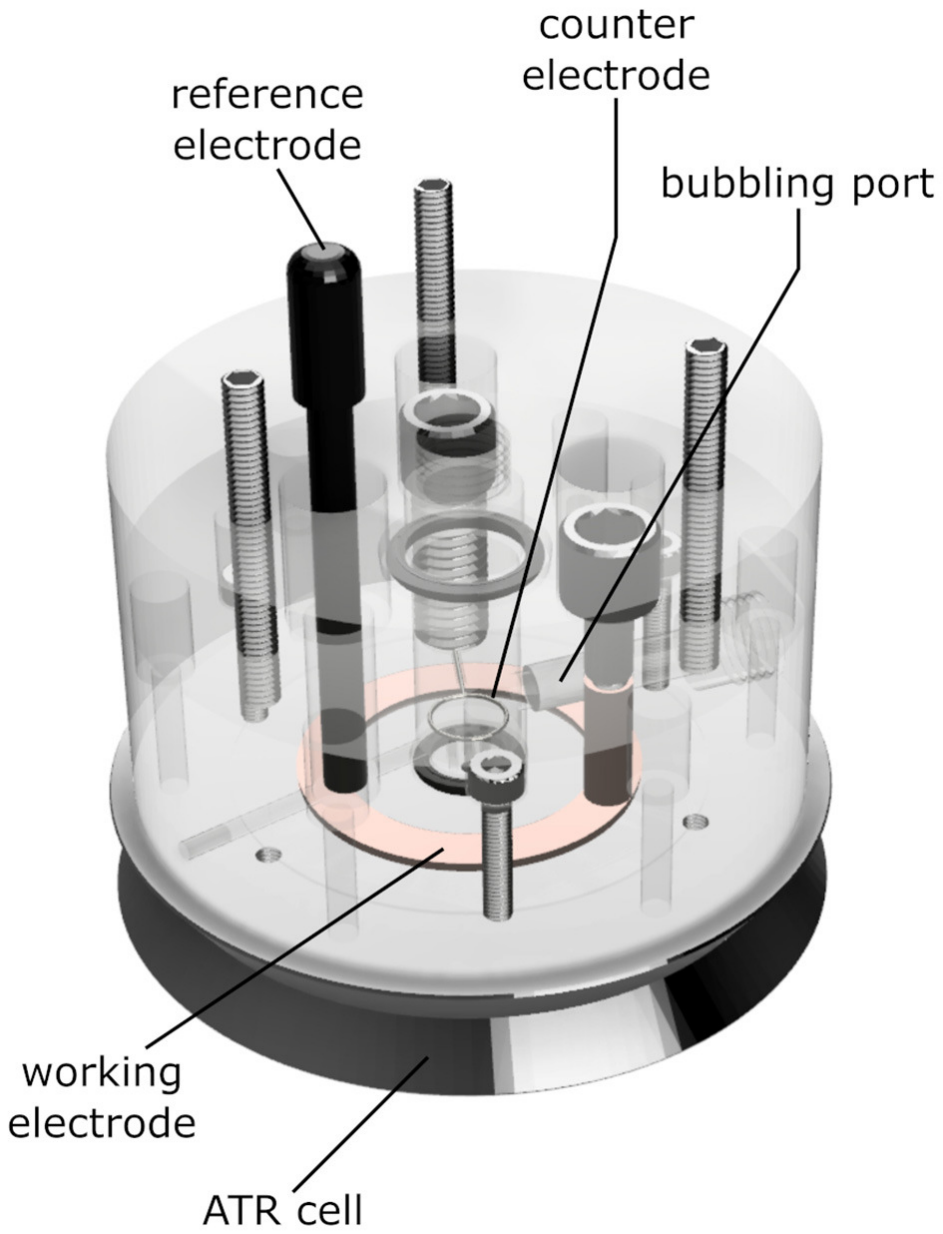
Schematics of the ATR electrochemical cell used in the experiments. The ATR unit is a three-reflection Si crystal. The permeable gold mesh used as a working electrode contacts the copper ring highlighted in the drawing. The sample is placed on top of the mesh, over the round Si crystal at the center of the picture. An 8 kDa dialysis membrane between the sample and the electrolyte prevents sample loss and excessive swelling. The gas inlet allows saturating the electrolyte and the sample with CO gas. The body of the cell is made out of PMMA, the O-rings are butyl rubber. The materials directly contacting the sample are a Kapton™ tape insulator, gold, the Si crystal itself, and the cellulose dialysis membrane.

We constructed an electrochemical cell (depicted in Figure 2) that allows ATR/FTIR measurements while purging a buffer reservoir on top of the sample with gas, basing our design on previous works (Dodia et al., 2013). The cell consists of a transparent acrylic body that can be screwed onto a Silicon ATR cell (Pike Technologies, WI, United States). The body of the cell has holes for a buffer reservoir, an Ag/AgCl reference electrode, an annular copper working electrode, a platinum counter electrode, and a gas-bubbling port.

The ATR crystal and its stainless-steel base were insulated using 95 μm thick Kapton tape (DuPont, DE, United States) in an annular shape. We deposited 5 μl of reconstituted C*c*O (15–20 μM, corresponding to 20–27 ng of protein) onto a 9 μm gold mesh (Goodfellow, PA, United States), adhered on top of the Si reflection element of the ATR setup. All experiments were performed anaerobically (*vide infra*). The sample was dried while monitoring the water bands of its absolute spectra. For samples measured in excess aqueous solution, we placed an 8 kDa dialysis membrane atop of the sample directly after drying to avoid excessive film swelling and subsequent sample loss. Then, we mounted the cell on top of the assembly and filled it with 3 ml of electrolyte buffer, i.e., 50 mM phosphate (pH 8) with 300 mM NaCl and 40 μM mediators (Hellwig et al., 1998). For the samples in D_2_O, we deposited 2 μl of pure D_2_O on top of the dry sample and redried it to facilitate buffer substitution in the protein. We then placed the dialysis membrane atop this film and filled the reservoir with D_2_O buffer [50 mM phosphate at pH^*^ 7.9 (Krazel and Bal, 2004), with 300 mM NaCl and 40 μM mediators]. We connected the electrodes in the cell to an Autolab potentiostat (Metrohm, Germany) and set the potential to −500 mV vs. Ag/AgCl while slowly purging the buffer solution with CO. The Ag/AgCl reference electrode was stored in saturated KCl before and after use. CO binding was performed over a period of about 1 h. The CO absorption was stable over 12 h; after which, a background spectrum was recorded at a reducing potential of −500 mV vs. Ag/AgCl. For the redox titration, the electrode potential was increased in steps of 25 mV every 450 s until a potential of +600 mV (vs. Ag/AgCl) was ultimately reached.

### FTIR Spectroscopy

All infrared absorption data were recorded on a Vertex 70 spectrometer, equipped with an MCT detector (Bruker, Germany). The spectrometer with the electrochemical cell was placed in a vinyl anaerobic chamber (COY lab products, MI, United States). Spectra were recorded in the frequency range of 4000–800 cm^−1^ at a spectral resolution of 2 cm^−1^ with at least 300 coadditions.

First, we monitored the binding of CO to C*c*O by recording the characteristic vibrational band of the C≡O stretching vibration that peaks at 1,964 cm^−1^ at −500 mV vs. Ag/AgCl at pH 8 (Mitchell et al., 1996). After performing the redox titration, we measured reduced-minus-oxidized spectra belonging to the two states identifiable from the analysis of the CO band shift in its fingerprint region, i.e., 1,950–1,980 cm^−1^ (Dodson et al., 1996; Mitchell et al., 1996; Iwaki and Rich, 2007). The only correction applied to our spectra was a Lorentzian Fourier filter, excluding components broader than 150 cm^−1^, acting essentially as a baseline correction (Supplementary Figure 1). The final difference spectra show characteristic protein bands, cofactor bands, as well as the CO rebinding with its band shift.

### Preparing the Crystal Structure for Electrostatic Energy Computations

The crystal structure of *Rs*C*c*O [PDB code: 2GSM (Qin et al., 2006)] was used and supplemented by hydrogen atoms, using CHARMM (Brooks et al., 2009). The atomic charges of the cofactors (heme a, heme a_3_, Cu_A_, and Cu_B_) were retrieved from previous work (Woelke et al., 2013). For the protein moiety, atomic partial charges were taken from the CHARMM force field. The CO ligand at heme a_3_ was placed using a structural overlay with the heme a_3_ of the *Bt*C*c*O crystal structure as shown in Figure 1 [PDB code: 3AG1 (Muramoto et al., 2010)]. The environment of the BNC in *Bt*C*c*O is virtually identical to the one of *Rs*C*c*O (Supplementary Figure 2). In the bovine structure, the Fe-C-O angle is 169°. This angle is nearly identical to the angle of 170° in the myoglobin crystal structure from sperm whale [PDB code: 3E5O (Tomita et al., 2009)]. The distances of Fe–C and Fe–O in *Bt*C*c*O are d_Fe−C_ = 1.67 Å and d_Fe−O_ = 2.40 Å, respectively. They differ slightly from the values in the myoglobin crystal structure, which are d_Fe−C_ = 1.88 Å and d_Fe−O_ = 2.99 Å (Tomita et al., 2009). In addition, we modeled an idealized geometry of CO bound to heme a_3_, with a Fe–C–O angle of 180° and d_Fe−C_ = 1.70 Å. The side chain of E286 is in the “down” conformation in the crystal structure, pointing away from the propionic side chain of heme a_3_, PRDa_3_. We performed energy minimization for the side chain of E286 to obtain a chemically reasonable structure in this case.

### Electric Field Computation Based on Crystal and Modeled Structures

We compared different redox states (oxidation of heme a or both heme a and Cu_A_) and protonation equilibria. In the following, the electric field at a point $\vec{r}$ generated by point charges *q*_i_ in positions $\vec{r_{i}}$ is defined as:

(1) $$\begin{array}{r} {\vec{E}}_{theory}\left(\vec{r}\right)=\frac{1}{4\pi \varepsilon_{r}\varepsilon_{0}}\overset{N}{\underset{i}{\sum }}q_{i}\frac{\vec{r}-{\vec{r}}_{i}}{|\vec{r}-{\vec{r}}_{i}{|}^{3}} \end{array}$$

where ε_0_ is the vacuum permittivity, with ε_0_ = 8.85·10^−12^ C/Vm and ε_r_ is the dielectric constant in the vicinity of the probe, which was set to ε_r_ = 4.0 (Kieseritzky and Knapp, 2008; Meyer and Knapp, 2015). In the present application, the positions of the hydrogen atoms were added to the crystal structure of C*c*O with HBUILD of CHARMM (Brooks et al., 2009) and energy minimized for oxidized cofactors (${\text{Cu}}_{\text{A}}^{2+}$, Fe^3+^ of both heme a and heme a_3_, ${\text{Cu}}_{\text{B}}^{2+}$), with most residues in standard protonation states, except for deprotonated K354 and protonated D407. The histidine tautomeric states were determined as in previous works (Woelke et al., 2013). The hydrogen atoms of heme a, the BNC, E286, the propionates of ring D (PRDa_3_) and ring A (PRAa_3_) at heme a_3_, as well as the histidines H333 and H334 that ligate Cu_B_, were energy minimized for the different charge states (Supplementary Figure 3). The geometry of all other atoms was not optimized. The atomic point charges *q*_i_ of these molecules vary according to the redox state of the enzyme, and it was necessary to consider them in order to compute the change of electric field in the transition between oxidized and reduced states. The change in the electric field at the CO in the direction of the CO bond, given by the unit vector $\vec{p}$, is described by:

(2) $$\begin{array}{r} \vec{p}\cdot △\vec{E}\left(\vec{r}\right)=\vec{p}\cdot \left[{\vec{E}}_{ox}\left(\vec{r}\right)-{\vec{E}}_{red}\left(\vec{r}\right)\right], \end{array}$$

where $\vec{r\;}$corresponds to the center of the CO bond.

Considering water molecules explicitly in computations of electrostatic energies can pose modeling challenges. Therefore, water is usually considered implicitly by filling its bulk volume with a dielectric medium of ε_r_ = 80. The crystal water molecules were not removed in our electrostatic computations, and their atoms were not subjected to charge-state-specific geometry optimization. In the presence of explicit water, its volume can be treated as the protein volume, using a dielectric constant of ε_r_ = 4.0. For the membrane volume, the same value of the dielectric constant is appropriate. The outer solvation layer of C*c*O is more than 22 Å away from the CO probe, such that the dielectric constant is also in this part ε_r_ = 4.0. Hence, a homogeneous dielectric medium can be used to describe the redox-induced variation of electrostatic field at the CO ligand of heme a_3_.

The changes of the electrostatic field at CO ligated to heme a_3_ are mainly governed by the variation of the atomic charges of heme a with its redox state. Since the charge changes are distributed over many heme atoms, only moderate conformational changes may occur as a result of the change in the heme a redox state, justifying why only specific hydrogen atoms (which are generally more mobile) were geometrically optimized. In the same spirit, using only geometry optimization and no molecular dynamics simulation, computations of heme redox potential were performed for artificial cytochrome b, with an accuracy of 20 mV (Popović et al., 2001). The agreement with experimentally measured heme redox potential is similar also for large proteins (Voigt and Knapp, 2003).

## Results

### Response of the C≡O Stretching Vibration to Changes in Redox Potential

**Figure 3:**
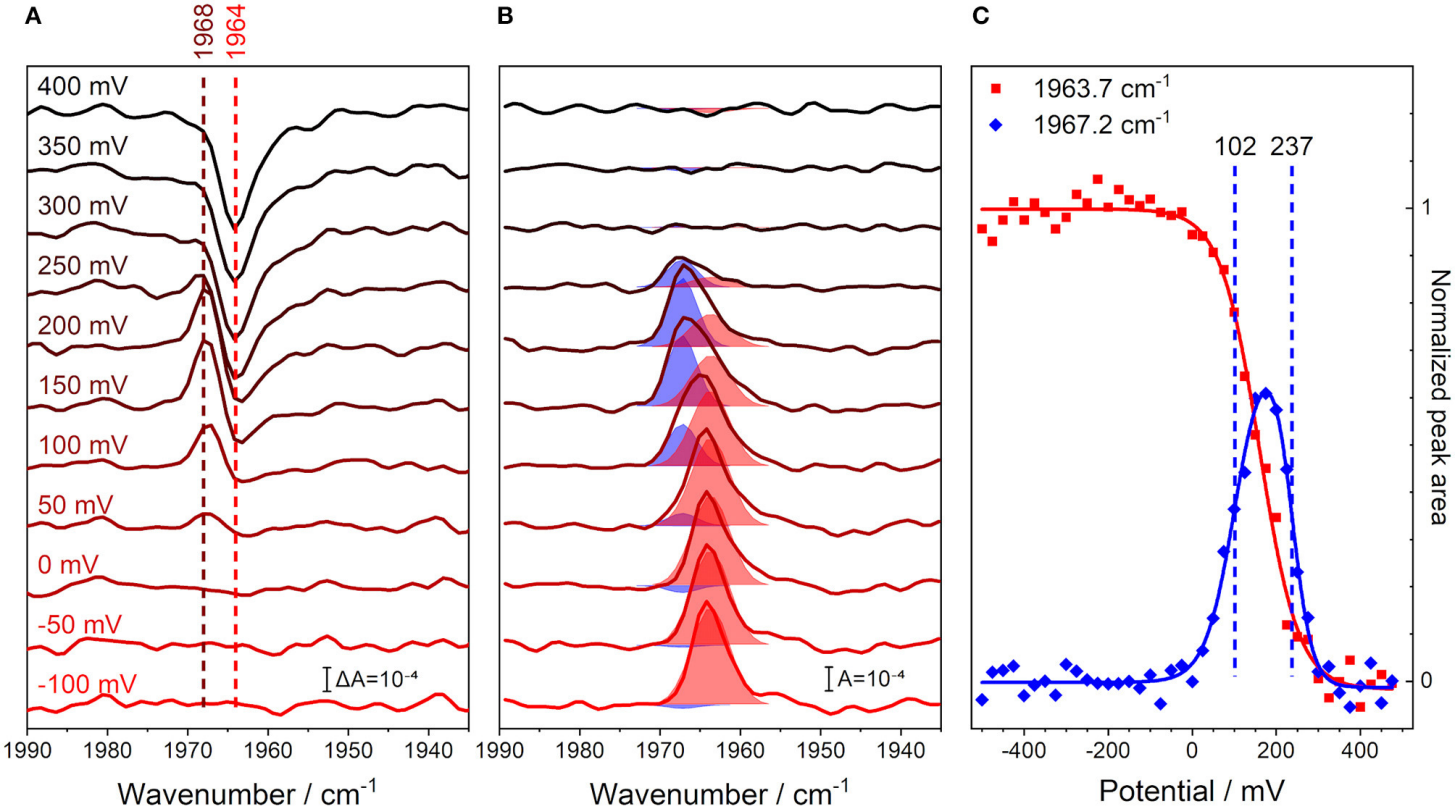
Monitoring the C≡O stretching band of iron-bound carbon monoxide in C*c*O and its unbinding from the enzyme through a potentiometric titration in H_2_O buffer. All potential values are given against the Ag/AgCl reference electrode. **(A)** Potential-resolved difference spectra of oxidized-reduced C*c*O from −100 (red) to +400 mV (black), background taken at −500 mV. The number of spectra is cut from the full potential range and reduced for clarity to one every 50 mV step. The peak positions for the initial shift of the CO band (brown), as well as the original CO peak position (red), are marked with dashed lines. **(B)** Potential-resolved absolute spectra of reduced-oxidized C*c*O obtained by subtracting the average of 3 spectra under oxidizing conditions (+500 to +550 mV). The spectra are indicative of the same potentials as in the left panel. The fits to the two CO bands are shown as shaded areas. The fits were obtained by fitting Voigtians with 20% Lorentzian and 80% Gaussian to all the spectra in this data set. Once the spectral fit converged, determining the peak positions and FWHMs, these parameters were maintained for all spectra, while the peaks' amplitudes were allowed to vary. **(C)** Full potential traces of the C≡O stretching peaks' integrals; steps of 25 mV from −500 to +550 mV. These data are proportional to the concentration profiles of the species. The peak areas at 1963.7 (red) and 1967.2 cm^−1^ (blue) are taken directly from the fit parameters calculated from the central panel and are shown as scatter plots. The sigmoidal fits of the peak area traces are shown as solid lines. The calculated midpoint potentials of the 1967.2 cm^−1^ peak fit function are displayed as dashed vertical lines.

We recorded an FTIR spectrum of fully reduced, CO-inhibited C*c*O at an electric potential of −500 mV vs. Ag/AgCl, which was used as a reference for the following spectra. Then, the electrode potential was increased in steps of 25 mV, and a series of potential-induced difference spectra were acquired in the range of the C≡O stretching vibration (Figure 3A). A positive band at 1,968 cm^−1^ evolves in the range of +50 to +200 mV. This feature develops into a single negative peak at 1,964 cm^−1^ for potentials higher than +300 mV. Such behavior indicates the presence of three different redox species.

In order to analyze this redox behavior rigorously, the potential-dependent difference spectra were transformed into absolute spectra, where the total contributions from the observed states are readily visible (Figure 3B). This data set was fit, using two Voigtian components with peak maxima at 1963.7 and 1967.2 cm^−1^. The blueshift of the CO peak is characteristic of the formation of the R_2_CO state (Dodson et al., 1996; Iwaki and Rich, 2007), i.e., the state in which the BNC is reduced, but the other metal centers in C*c*O are oxidized (Brzezinski and Malmström, 1985). The identifiable states are: (I) a state where CO is initially bound under reducing conditions [R_4_CO with ν(C≡O) = 1963.7 cm^−1^], (II) a state in which the majority of the sample has undergone the CO band shift [R_2_CO with ν(C≡O) = 1967.2 cm^−1^], and (III) a state in which the CO ligand finally dissociates from the BNC (O) (Dodson et al., 1996; Cooper and Brown, 2008).

Plotting the relative abundance of the two CO-inhibited species against the applied electrode potential illustrates the depletion and formation of the R_4_CO, R_2_CO, and O states (Figure 3C). The analysis of the potential-dependent traces provides midpoint potentials of +102 ± 11 mV for the R_4_CO → R_2_CO transition and +237 ± 6 mV for the R_2_CO → O transition, which we will discuss below (these values are almost invariant to the specific fit procedure; see Supplementary Figure 4). It should be noted that the second transition involves the dissociation of the CO ligand. However, rebinding of the CO ligand to heme a_3_ was a reversible process under the conditions and at the time scales of the experiment (Supplementary Figure 5). Even though the R_2_CO state is stable and exhibits an easily identifiable CO band, it is not possible to accumulate a pure R_2_CO state under any conditions. In fact, the R_2_CO state only represents 60% of the total species at its maximum of +175 mV (Figure 3C). The O state is accumulated at highly positive electrode potentials and is indicative of complete oxidation of the BNC. Evidently, it is impossible to exclude the contribution of a one-electron-reduced BNC component in the O state since the unbound CO probe cannot report any longer on the electronic state of its surroundings.

The frequency of the C≡O vibration can be used to quantify electrostatic changes in the local environment *via* the VSE (Park et al., 1999; Boxer, 2009), under the condition that the redox state of heme a_3_ remains unchanged. As such, the spectral shift from 1963.7 to 1967.2 cm^−1^ can be interpreted in terms of an altered electric field projected on the CO bond upon the R_4_CO → R_2_CO redox transition. In the case of C*c*O, the CO ligand adopts a well-defined conformation when bound to heme a_3_ (as indicated by its narrow bandwidth), with an angle given by the BNC coordinates (Muramoto et al., 2010). Thus, one can interpret the frequency shift of the CO probe using the linear VSE equation:

(3) $$\begin{array}{r} \Delta \nu =|\Delta \vec{\mu }|\cdot \Delta E \end{array}$$

where $|\Delta \vec{\mu }|=2.4/f$ cm^−1^/(MV/cm) is the Stark tuning rate of CO bound to a heme iron (Park et al., 1999) and $\Delta E=\vec{p}\cdot \Delta \vec{E}$ is the change in the local electric field projected onto the CO bond axis $\vec{p\;}$ (see Supplementary Figure 6 for the convention on the electric field direction) (Park et al., 1999; Suydam and Boxer, 2003). The factor *f* is the local field correction and is required when experimentally ascertaining the local electric field in a protein. This parameter is a result of the specific experimental design of vibrational Stark spectroscopy which provided the Stark-tuning rate, and has been suggested to be ~2 in recent work (Fried and Boxer, 2015; Schneider and Boxer, 2016). Using our experimentally determined blueshift of Δν = +3.5 cm^−1^ for the redox titration, we infer that the change in electrostatic field experienced by the CO probe along its bond axis is Δ*E* = +2.9 MV/cm.

### Evaluating Protonation Changes Upon Reduction by Electrostatic Energy Computation

We performed electrostatic computations to disentangle the various contributions to the electric field changes. The calculated electric field changes projected onto the CO bond ($\vec{p}\cdot \Delta \vec{E}$) are compared to the electric field change of +2.9 MV/cm as derived from the measured blueshift of +3.5 cm^−1^ upon oxidation of C*c*O. The experimentally determined electric field is a result of the individual contributions resulting from the change in oxidation states of the cofactors, as well as the protonation states of E286, the propionates of heme a_3_ (PRDa_3_ and PRAa_3_), and the residues H333/H334 and Y288. Here, we used electrostatic energy computations by solving the Poisson-Boltzmann equation to evaluate pKa values. This approach allows isolating the different contributions to the electric field changes. Alternatively, MD simulations with polarizable force fields were successfully used to match the electrostatics determined from MD simulation to experimental results (Welborn and Head-Gordon, 2019; Wu et al., 2020).

The charge changes displayed in Table 1 are organized such that the charge increases by one elementary unit for all the considered transitions. The possible contributions of H333, H334, and Y288 are so large that any change in their protonation states can be ruled out for the present experiments. Our electrostatic computations also account for the two different side-chain conformations of E286. It has been discussed that this flexibility allows E286 to act as a proton valve near the entrance of the D-pathway (Belevich et al., 2006; Kaila et al., 2008, 2011; Woelke et al., 2013). In the reported crystal structures of wild-type C*c*O, E286 is in the “down” conformation, where the carboxylic group points toward the entrance of the D-pathway (Supplementary Figure 3) (Yoshikawa et al., 1998). However, in the structure of the N131D mutant of *Pd*C*c*O, E278 (equivalent to E286 in *Rs*C*c*O) adopts an alternative rotamer conformation with the carboxylic acid group pointing toward the P side of the membrane (Dürr et al., 2008). We modeled the alternative “up” conformation of protonated E286, as suggested by molecular dynamics simulations (Kaila et al., 2008). We also considered the two charge-neutral protonation states, namely, E286H^1^ and E286H^2^ with hydrogen atoms at O^1^ and O^2^, respectively.

**Table 1:** Electrostatics computations.

**Group**	**Transition**	**Fe–C–O angle 169^**°**^**	**Fe–C–O angle 180^**°**^**
heme a	Fe^2+^ → Fe^3+^	0.82	0.83
Cu_A_	Cu^1+^ → Cu^2+^	0.13	0.07
E286 (down)	E286^−^ → E286H^1^	−1.61	−1.49
E286 (down)	E286^−^ → E286H^2^	−1.50	−1.38
E286 (up)	E286^−^ → E286H^1^	−2.24	−2.10
E286 (up)	E286^−^ → E286H^2^	−1.94	−1.83
PRDa_3_	PRD^−^ → PRDH^1^	0.52	0.19
PRDa_3_	PRD^−^ → PRDH^2^	0.79	0.52
**PRAa**_**3**_	**PRA**^−^→**PRAH**^**1**^	**2.17**	**1.69**
**PRAa**_**3**_	**PRA**^−^→**PRAH**^**2**^	**1.85**	**1.37**
Y288	Y288^−^ → Y288H	−7.28	−6.97
H334	H334 → H334^+^H^δ^	−11.45	−11.71
H333	H333 → H333^+^H^δ^	−11.87	−12.06

*Electric field changes of CO bound to the iron of heme a_3_ and Cu_B_ are computed at the CO-bond center projected onto the CO bond $\vec{p}$. The values for $\vec{p}\cdot \Delta \vec{E}$ are given in units of MV/cm. While CO is bound, heme a_3_ and Cu_B_ are kept in the reduced state with Fe^2+^ and ${\text{Cu}}_{\text{B}}^{+}$. The electric field changes are induced by oxidation of heme a and Cu_A_, and changes in the protonation states of E286, the propionic acids PRDa_3_ and PRAa_3_, as well as of the histidines H333 and H334. The two histidines, ligated to Cu_B_, are doubly deprotonated in the deprotonated state. Furthermore, two orientations of E286 were considered with the proton attached either at O1 or O2 of the carboxylic acids. The computed electric field changes that are compatible with the measured value of 2.9 MV/cm are highlighted by bold digits*.

Computing the oxidation of heme a and Cu_A_ yielded electric field changes of +0.82 and +0.13 MV/cm, respectively (Table 1). Upon oxidation of heme a, experimental data suggest a protonation of a heme a_3_ propionate, and corresponding computed electric field changes range from +0.52 to +2.17 MV/cm (Table 1). Initially, the propionate side chains of both hemes have been considered as candidates for acting as the PLS (Behr et al., 1998). More recent studies have favored the propionates of heme a_3_ (Kaila et al., 2011; Sezer et al., 2017). Adding the contributions of heme a and Cu_A_ oxidation and PRDa_3_ protonation yields a result which is in moderate agreement (i.e., a similar sign but deviant magnitude) with the measured electric field, regardless of the CO geometry. Instead, considering the oxidation of heme a and Cu_A_ together with the protonation of PRAa_3_ is in line with our experimental data (+3.12 or +2.59 MV/cm, depending on the modeled CO angle vs. +2.9 MV/cm of the experimental estimate). In contrast, protonation changes of E286, H333, and H334 lead to negative field changes and disagree with the present experiment.

### Protein Response to Changes in the Applied Electric Potential

**Figure 4:**
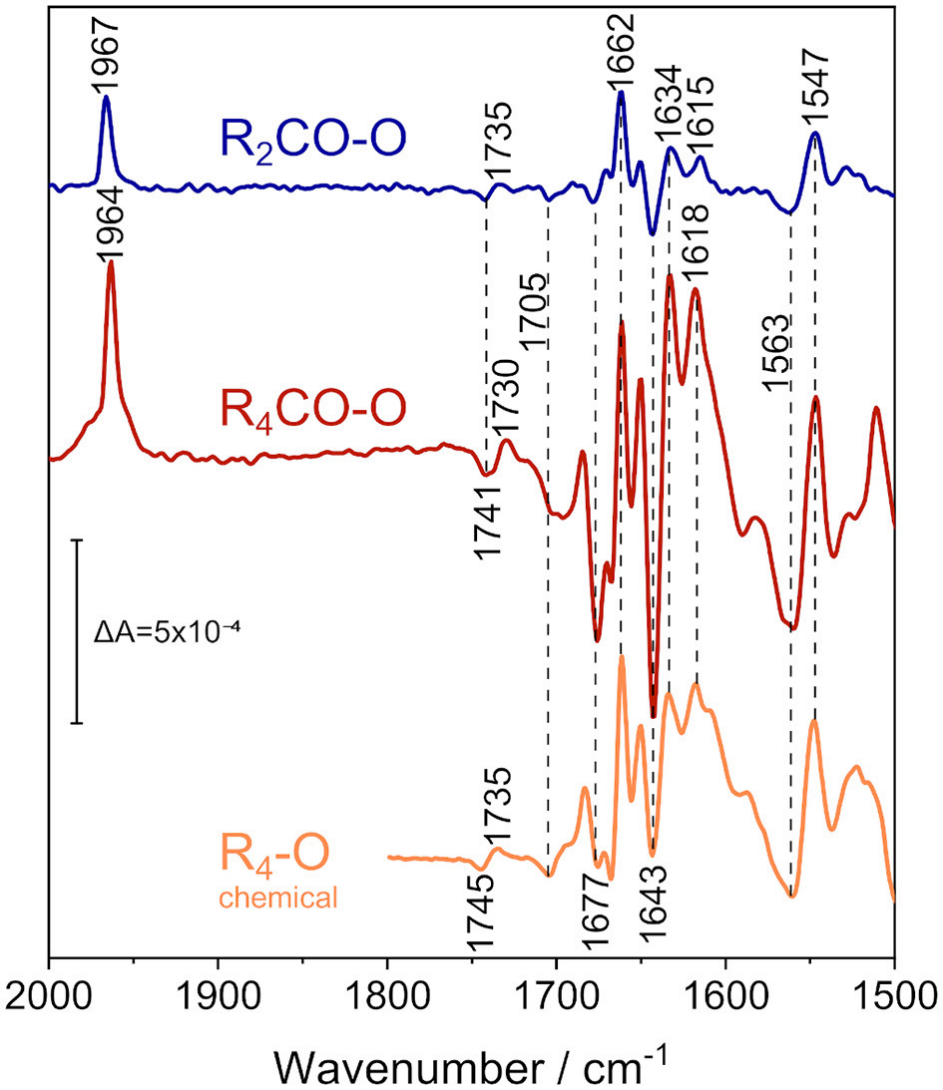
ATR/FTIR difference spectra of the reduced states of CO-bound *R. sphaeroides* C*c*O reconstituted in ^13^C-isotopically labeled lipids in D_2_O electrolyte buffer. Applying a potential of +180 mV vs. Ag/AgCl isolates the 2-electron-reduced state (R_2_CO, top spectrum, in blue), while fully reducing the enzyme at −800 mV vs. Ag/AgCl results in the 4-electron-reduced species (R_4_CO, middle, in red). CO and amide IR regions are shown. The data is compared to the perfusion-induced spectrum of fully reduced C*c*O in H_2_O, in the absence of CO (bottom spectrum, in orange, data taken from Nyquist et al., 2001). Band positions are labeled, and positions that do not differ between the states are highlighted by vertical dashed lines. The vertical marker shows the difference absorbance scale.

Analyzing CO-inhibited C*c*O, we pinpointed the transition potentials between the R_4_CO, R_2_CO, and O states. After the equilibration in the O state, we applied rapid potential jumps, leading to the R_2_CO and R_4_CO states. Each potential jump was followed by a longer equilibration period of ~30 min at the oxidizing potential. Given the different choice of background, it is important to remember that the following data show the process opposite to Figure 3, i.e., protein reduction instead of oxidation. The steady-state spectra of Figure 4 show that CO easily rebinds to the protein after oxidation, if reducing conditions are provided (for more details, see Supplementary Figure 5).

We recorded FTIR difference spectra of CO-inhibited C*c*O reconstituted in DPPC. Instead of the native lipid, we used the ^13^C_40_-labeled isotopomer of DPPC (Hübner and Mantsch, 1991) to avoid any possible spectral overlap with the C=O stretching vibration of glutamic acid and aspartic acid, absorbing at around 1,740 cm^−1^. Furthermore, the ester carbonyl bands of DPPC can shift with electric field changes (Zawisza et al., 2007). FTIR difference spectroscopy has been conducted with the sample immersed in D_2_O buffer to avoid spectral interference from the water bending vibration at 1,640 cm^−1^ (for a comprehensive overview of the differences that these isotope substitutions entail, see Supplementary Figures 7–9).

In these experiments, we used the FTIR spectrum of oxidized C*c*O (O) as a reference. The O state spectrum was accumulated at +600 mV. A rapid potential jump from +600 to +180 mV was applied to the sample. This resulted in the formation of the R_2_CO state, and the R_2_CO-O difference spectrum was recorded (top spectrum in Figure 4). In R_2_CO, the BNC is reduced, but heme a and Cu_A_ are oxidized. Since CO was present in our spectroelectrochemical cell, the reduction of the BNC resulted in immediate binding of CO to heme a_3_, as evident from the band at 1,967 cm^−1^.

The electrochemically-induced FTIR difference spectrum between the fully reduced and oxidized states (R_4_CO–O, Figure 4, middle spectrum) was recorded at −800 mV. It is noted that this spectrum is almost identical to the R_4_-O spectrum of the fully reduced state generated by the presence of the chemical reductant sodium dithionite (Nyquist et al., 2001) (Figure 4, bottom spectrum), despite the presence of the CO ligand in the former (as evident from the band at 1,964 cm^−1^). The strongest bands have been assigned to cofactor redox transitions (Babcock and Salmeen, 1979; Woodruff et al., 1981; Kozuch et al., 2013). The insensitivity of the difference bands to H/D agrees with this assignment. The only exception is the sigmoidal band pair at 1,741/1,730 cm^−1^. This band feature was assigned to the change in hydrogen bonding of the C=O group of the carboxylic side chain of E286 (Nyquist et al., 2001), which absorbs at 1,745/1,735 cm^−1^ in H_2_O (see R_4_-O spectrum in Figure 4). C*c*O from *Paracoccus denitrificans* (*Pd*C*c*O) exhibits the same vibrational changes in potentiometric titrations (Hellwig et al., 1998; Gorbikova et al., 2006).

A negative band at 1,705 cm^−1^ appears in both the R_4_CO and R_2_CO states. This band has been tentatively assigned to the C=O stretching vibration of a carboxylic side chain of an aspartic or glutamic acid (Hellwig et al., 1999b). Also, the propionic acid side chain of heme a_3_ in the ba_3_ enzyme from *Thermus thermophilus* was proposed (Koutsoupakis et al., 2011). The negative band indicates that such carboxylic side chain is protonated in the oxidized state of C*c*O and deprotonated upon reduction of the BNC in the R_2_CO and R_4_CO states.

Bands between 1,700 and 1,620 cm^−1^ are either due to peptide backbone vibrations (amide I) or to vibrational bands of the redox cofactors. The first of such features is the negative band at 1677 cm^−1^, possibly indicative of amide I (Dodia et al., 2013) or of propionic acid A of heme a_3_ (Behr et al., 1998, 2000). This band is weak in the R_2_CO state and may well be due to contamination by the R_4_CO state, as apparent from our analysis of the potential dependence using bound CO as a sensor (Figure 3C). The band position also does not change upon H/D exchange (Supplementary Figure 7). Given these considerations, we favor the assignment to the ν(C=O) of one of the hemes' propionic acids. The intense positive peak at 1,662 cm^−1^ originates in the O → R_2_CO transition, since its intensity and frequency barely change upon transition to the R_4_CO state. Thus, this positive feature probably correlates only with heme a_3_ reduction and is not the peak-shifted counterpart of the negative feature found at 1,677 cm^−1^. The band has previously been assigned either to amide I (Gorbikova et al., 2006), or to the heme a_3_ formyl ν(C=O) in the cofactor's reduced state (Heibel et al., 1993; Hellwig et al., 1999a,b; Nyquist et al., 2001). This band is also not influenced by H/D exchange in our experiment, and it appears to show a single unconvoluted peak. Therefore, it is unlikely to be due to amide I and is, thus, assigned to heme a_3_ formyl.

The majority of difference bands between 1,700 and 1,500 cm^−1^ have been assigned to heme a and heme a_3_, facilitating the direct readout of the cofactor redox states. The most intense negative peak at 1,643 cm^−1^, for example, is found mainly in the R_4_CO state. It was previously assigned to the formyl groups of the oxidized heme a (Babcock and Salmeen, 1979; Hellwig et al., 1999a; Kozuch et al., 2013). Surprisingly, this band is also present in the R_2_CO spectrum (Figure 4, top spectrum), albeit with a less intense negative peak. It is likely that the feature at 1,643 cm^−1^ correlates with heme a reduction in the R_2_CO → R_4_CO transition. However, even when considering R_4_CO contamination in the R_2_CO state (*vide supra*), we cannot exclude the possibility that this band originates from the R_2_CO state. Reduced heme a exhibits a characteristic band, which is the ν_10_(C=C) vibration at 1,634 cm^−1^ (Heibel et al., 1993; Kozuch et al., 2013); for which, the same considerations apply. Another band that changes in absorbance in the two reduced states can be identified at 1,618 cm^−1^ in the R_4_CO spectrum and at 1,615 cm^−1^ in the R_2_CO spectrum. These peaks are assigned to the vinyl ν(C=C) of reduced heme a_3_ (Heibel et al., 1993; Hellwig et al., 1999a; Dodia et al., 2013). The small peak shift of 3 cm^−1^ indicates that the heme a_3_ ring reacts in response to the change in the internal electric field as a result of the reduction of heme a.

The low-energy region between 1,570 and 1,540 cm^−1^ shows mainly two strong bands, a broad negative band at 1,563 cm^−1^ and a positive one at 1,547 cm^−1^. The latter and the formyl band at 1,662 cm^−1^ are the only two bands that do not exhibit a strong change in absorbance between R_2_CO and R_4_CO, which links their appearance to heme a_3_ reduction. While isotopic labeling hinted toward the asymmetric carboxylate stretching mode ν_as_(COO^−^) of the deprotonated PRDa_3_ (Behr et al., 1998, 2000), resonance Raman experiments have shown that bands at similar positions can also be assigned to the heme a ν_11_ vibration (Heibel et al., 1993; Kozuch et al., 2013). The assignment of the negative band at 1,563 cm^−1^ is ambiguous as well: Resonance Raman studies assign this frequency to the ν_38x_ mode of oxidized heme a_3_ (Kozuch et al., 2013); however, the band could also be due to the ν_as_(COO^−^) mode of heme propionate(s) (Hellwig et al., 1999a,b) in the O state. In this scenario, proton transfer between heme propionic acid side chains in the O → R_2_CO transition would justify the presence of the 1,563 and 1,547 cm^−1^ bands in our spectra.

## Discussion

**Figure 5:**
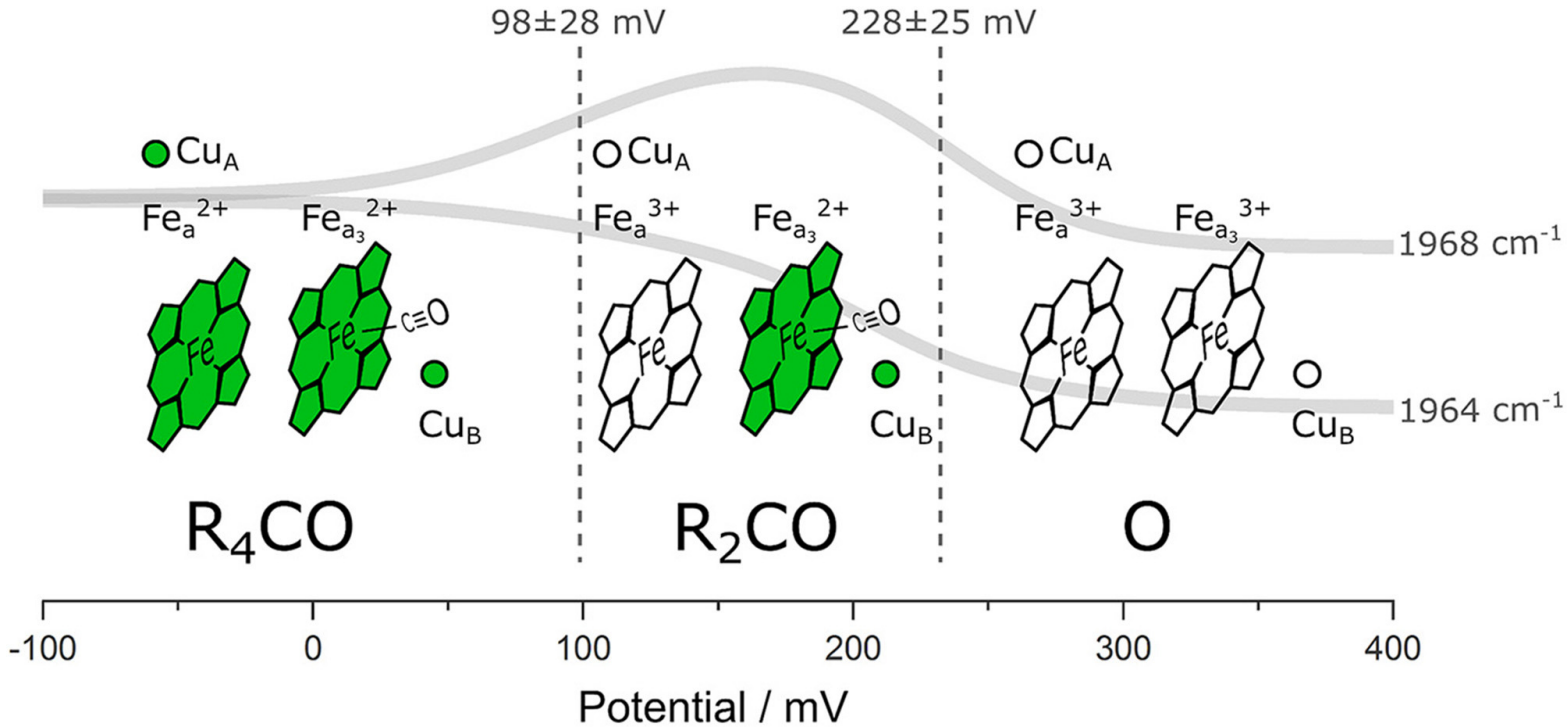
Schematic representation of the different C*c*O states distinguishable in our experiment. The four redox-active metal cofactors are figuratively depicted as reduced (green) or oxidized (transparent). The CO probe is shown in its bound states, and the global fit of the potential-dependent CO band intensity is shown as solid gray traces. Dashed vertical lines correspond to the midpoint potentials of the redox transitions. The identified states are labeled according to the nomenclature used in this work. The potential values are given against the Ag/AgCl reference electrode.

We performed ATR/FTIR spectroelectrochemistry on lipid-reconstituted C*c*O in the presence of CO. Exploiting the CO ligand as a VSE probe, we identified three states corresponding to different electrode potentials applied to the protein film (Figure 5): (I) the fully reduced state R_4_CO (in which all four metal centers are reduced), (II) the mixed-valence state R_2_CO with two electrons (reduced heme a_3_ and Cu_B_), and (III) the oxidized state O (all four metal centers are oxidized) in which CO is dissociated from the BNC. It was not possible to trap a pure R_2_CO state under our experimental conditions, and we can also not exclude a possible one-electron component in what we denote as the O state. It is important to notice that this hypothetical one-electron-reduced state, analogous to the E state of C*c*O under turnover conditions, would imply the partial reduction of Cu_B_ at the BNC (Belevich and Verkhovsky, 2008). However, all four redox centers of C*c*O are oxidized at the potentials higher than +300 mV vs. Ag/AgCl (ca. +500 mV vs. SHE) (Wilson et al., 1976). It is also evident that the midpoint potentials derived from sigmoidal fitting (102 and 237 mV, Figure 3C) and global fitting of the difference spectra (98 and 228 mV, Supplementary Figure 4A) are identical within the error margin (34 and 25 mV, respectively, Supplementary Figure 4A). Other studies (Gorbikova et al., 2006) also show a possible intermediate state by following oxidation of the cofactors through specific marker bands. In fact, the potential traces of the two CO peaks exhibit similar behavior as vibrational bands found in potentiometric titrations of *Pd*C*c*O in the absence of CO (Hellwig et al., 1999a; Gorbikova et al., 2006). The difference between the midpoint potentials of CO-bound C*c*O and the potentials relative to the CO-free enzyme is small [23 mV (Dodson et al., 1996)]. However, the midpoint potential of the R_4_CO → R_2_CO transition is higher in *Rs*C*c*O than reported (Hellwig et al., 1999a; Gorbikova et al., 2006). We relate these differences to the probe's selectivity, allowing us to observe electrochemical transitions whose trace is otherwise very convoluted.

We quantified the local electric field by measuring the frequency shift of the CO vibration and by performing electrostatic computations. We derived a +2.9 MV/cm change in the total electric field projected on the CO bond, induced by the redox transition from R_4_CO to R_2_CO (in which heme a and Cu_A_ are oxidized). As elegantly elaborated by Kaila et al. (2010), electric fields can orient charged and polar residues (including water molecules) to generate a proton pathway with low energetic barriers. Thus, the quantification of the electric field magnitude achieved here represents an essential requirement to gauge the driving force of proton transfer in the vicinity of the BNC.

The primary cause of the Stark shift can be attributed to the redox transition of heme a, which contributes with an electric field of +0.82 MV/cm projected onto the CO ligand according to the electrostatic calculations. In comparison, the simultaneous oxidation of the distant Cu_A_ accounts for a much smaller extent of +0.13 MV/cm. These redox transitions can induce further protonation and/or structural changes whose electrostatic contributions are shown in Table 1. Combining the electric field changes due to oxidation of heme a and Cu_A_ and simultaneous protonation of PRAa_3_ (+3.12 or +2.59 MV/cm for CO angles of 169° or 180°, respectively) agrees with the change in the electric field that was experimentally determined (+2.9 MV/cm). Comparing this result with the behavior of the band at 1,677 cm^−1^ in our spectra, we may refer to PRAa_3_ as a deprotonation site in the physiological O → R transition, most likely involving E286 as a relay site. Combining the oxidation of heme a with any other transition shown in Table 1 results in less agreement with our experimental findings. For example, the computed values of the change in the electric field related to changes in the protonation state of E286 are negative, which indicates that this transition does not take place. The E286 ν(C=O) peak shift between the oxidized state and the two reduced states corroborates this prediction. This observation also excludes the possibility of a protonation/deprotonation event, since the carboxylate group has symmetric and antisymmetric COO^−^ stretching vibrations that appear at around 1,570 and 1,400 cm^−1^ (Zscherp et al., 1999; Barth, 2007). The collected data hint at E286 being protonated in all the observed states. Even though our sample does not undergo catalytic turnover in the absence of O_2_, we think that the same assumptions apply to the physiological O and R_2_ states of C*c*O, as well as to the R_4_ state (Nyquist et al., 2003).

The major change that E286 undergoes upon reduction is a change in its hydrogen-bonding environment (Nyquist et al., 2004; Barth, 2007). It has been recently computed that E286 is connected to a water cavity when it adopts the “up” conformation (Son et al., 2017). In the “down” conformation, on the contrary, it is close to the terminal water molecule of the D-pathway [W6560 in oxidized *Rs*C*c*O crystals (Tomita et al., 2009)]. Our data suggest a picture in which E286 is hydrogen bonded either to one of such water molecules, or to some unidentified residue. The hydrogen bond is weakest in the oxidized state and gets progressively stronger when lowering the electrode potential [redshifts of the ν(C=O) peak are 6 cm^−1^ and 11 cm^−1^ for the R_2_CO and R_4_CO states, respectively]. Depending on the exact origin of this shift, the interpretation hints to E286 changing its conformation from “up” to “down” during oxidation of heme a, or *vice versa*. The hydrogen-bonding environment experienced by E286 may also depend on its conformation relative to the propionic acid side chain of heme a_3_. These findings add to the current knowledge (Nyquist et al., 2001, 2003; Heitbrink et al., 2002) and suggest that the reduction of heme a_3_ (happening after the first proton pumping step following the E state, under physiological conditions) already influences the hydrogen-bonding environment of E286.

Finally, the potentiostatic FTIR difference spectra in D_2_O show very few band shifts that are typical for protein backbone deuteration. Adding our interpretation and previous assignments to this fact, we suppose that almost all the assigned bands appearing in the window between 1,710 and 1,500 cm^−1^ in Figure 4 are either cofactor bands or representatives of “buried” backbone motifs. However, residues in the vicinity of the BNC which are not accessible to bulk solvent ought to be scarce when C*c*O cycles through reduced and oxidized states (Busenlehner et al., 2006). Our observations relate cofactor bands to the redox transition responsible for their appearance and are especially interesting when analyzing bands that have previously been ambiguous (Hellwig et al., 1999a,b; Dodia et al., 2013), as they indicate that the C=O-stretching features in the area between 1,710 and 1,640 cm^−1^ are most likely due to propionic acid or formyl bands. Moreover, bands below 1,710 cm^−1^ do not show frequency shifts between the two reduced states, with the vinyl mode of reduced heme a_3_ found at 1,615 and 1,618 cm^−1^ being the only exception. The exact reason for this shift is currently unknown, but it is reasonable to assume that the protonation state of PRAa_3_ can change the coupling mode of the ν(Cα=Cβ) of the same porphyrin ring, influencing its vibrational frequency. There are only two bands that correlate with heme a_3_ reduction, which are the bands at 1,662 and 1,547 cm^−1^. While it is difficult to assign the latter, the former is due to the formyl C=O stretching vibration of reduced heme a_3_. In fact, this is the only intense band at a frequency compatible with C=O groups among the ones that are associated with the transition at +237 mV, and it lacks a respective shifted band in the oxidized state. This feature may be representative of a strong perturbation of the environment close to the formyl side chain of heme a_3_ upon reduction (which takes place during the physiological E → R transition). It is not immediately clear whether the band at 1,643 cm^−1^ correlates with the same redox transition, or if it is indicative of a separate process involving only the formyl side chain of heme a. In general, our data show that the ligand interaction sphere of heme a_3_ is already disturbed in the O → R_2_CO transition, with possible propionate protonation/deprotonation events indicative of proton transfer close to the cofactor. This scenario would not involve heme a but only heme a_3_. Its formyl side chain may be responsive to these changes, resulting in the 1,662 cm^−1^ positive band. Adding this information to the probable protonation of PRAa_3_ upon heme a oxidation, our analysis supports the assignment of the proton-loading site to the propionates of heme a_3_ (Behr et al., 1998; Kaila et al., 2011; Sezer et al., 2017).

## Data Availability Statement

The original contributions presented in the study are included in the article/Supplementary Material, further inquiries can be directed to the corresponding author.

## Author Contributions

FB performed FTIR measurements, analyzed the spectroscopic data, and wrote the paper. JD constructed the CcO models and computed the electric field changes. JK, HM, and STS analyzed and discussed the data. E-WK and JH conceived the study. All authors discussed the results and contributed to the writing of the paper.

## Conflict of Interest

The authors declare that the research was conducted in the absence of any commercial or financial relationships that could be construed as a potential conflict of interest.

## Abbreviations

ATR: attenuated total reflection
BNC: binuclear center formed by heme a_3_ and Cu_B_
*Bt*C*c*O: cytochrome *c* oxidase from *Bos Taurus*
C*c*O: cytochrome *c* oxidase
DPPC, 1: 2-dipalmitoyl-sn-glycero-3-phosphocholine
E286: glutamate 286 of *Rhodobacter sphaeroides*
FTIR: Fourier transform infrared spectroscopy
O: state in which all redox cofactors are oxidized
PLS: proton loading site
PRAa_3_: ring A propionate of heme a_3_
PRDa_3_: ring D propionate of heme a_3_
*Pd*C*c*O: cytochrome *c* oxidase from *Paracoccus denitrificans*
R_2_, two-electron reduced state in which Cu_A_ and heme a are oxidized: while the binuclear center is reduced
R_2_CO: two-electron reduced state with carbon monoxide ligating heme a_3_
R_4_: fully reduced state in which all metal cofactors are reduced
R_4_CO: fully reduced state with carbon monoxide ligating heme a_3_
*Rs*C*c*O: cytochrome *c* oxidase from *Rhodobacter sphaeroides*
VSE: vibrational Stark effect.
