# Supplementary material for: Quantification of Local Electric Field Changes at the Active Site of Cytochrome c Oxidase by Fourier Transform Infrared Spectroelectrochemical Titrations
Source: Front Chem. 2021 Apr 27;9:669452. doi: 10.3389/fchem.2021.669452 (PMC8111224; doi:10.3389/fchem.2021.669452)
Supplement: Supplementary file 1 [file Data_Sheet_1.DOCX]

Supplementary Material


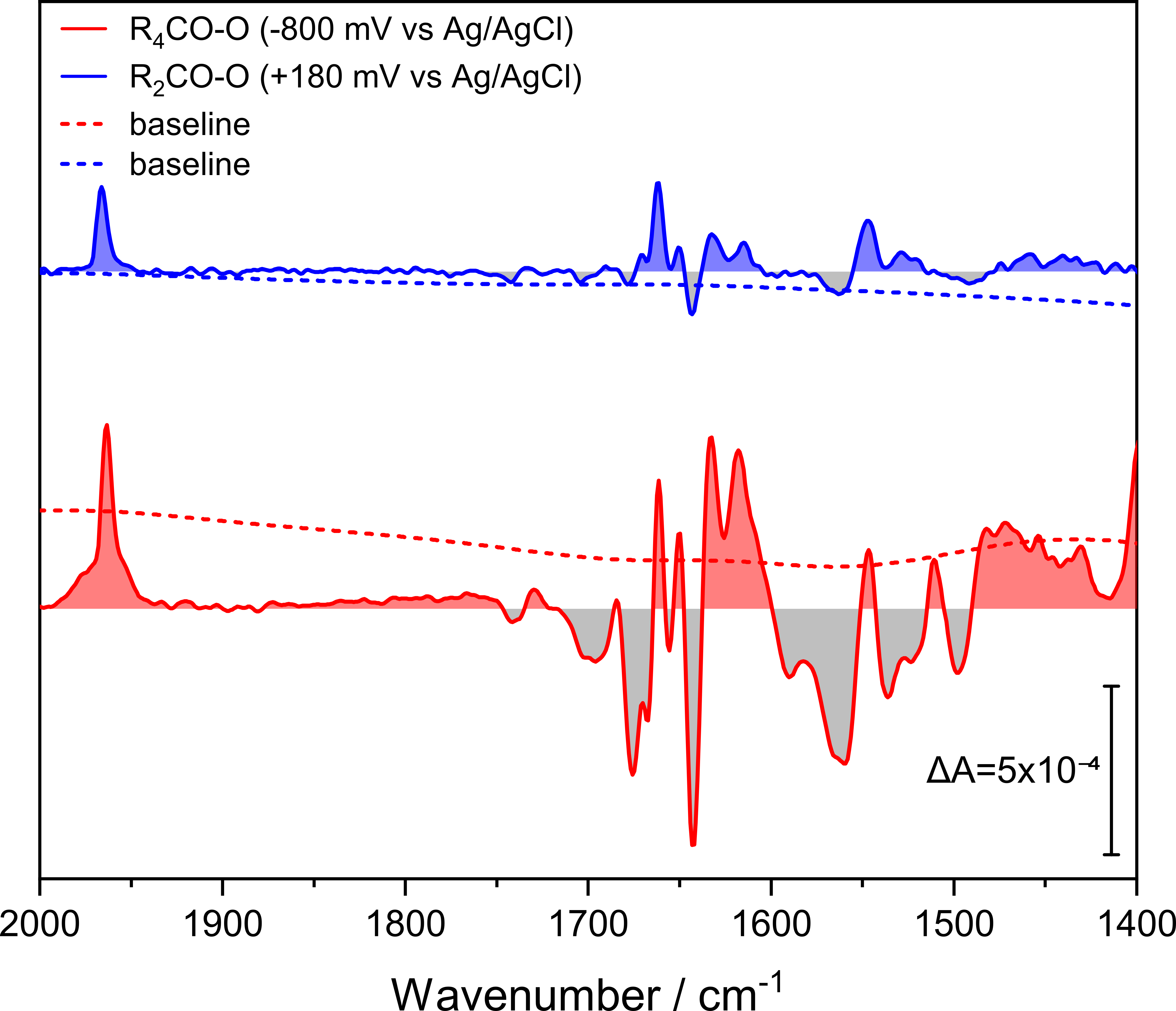


Figure S1. Steady-state difference spectra of the R_2_CO and R_4_CO states of CcO reconstituted in ^13^C isotopically labelled lipids in D_2_O electrolyte buffer (same as in Figure 4) and the subtracted baseline (dashed traces). The baseline was obtained by applying a low-pass Lorentzian Fourier filter with broadness of 150 cm^-1^ to the raw spectral data over the full spectrum (4000–800 cm^-1^).

Figure S2. CcO crystal structures from *Bos taurus* (PDB code: 3AG1, carbon atoms in magenta) and *Rhodobacter sphaeroides* (PDB code: 2GSM, carbon atoms in white). The overlay shows heme a and heme a_3_ (A) as well as Cu_B_ and relevant amino acid sidechains (B). Note the similarity of structures, in particular heme a_3_ and the Cu_B_ binding site. Histidines H333, H334, and H284 coordinate Cu_B_ while tyrosine Y288 and glutamate E286 were suggested to serve as proton donors/ acceptors (*R. sphaeroides* nomenclature).


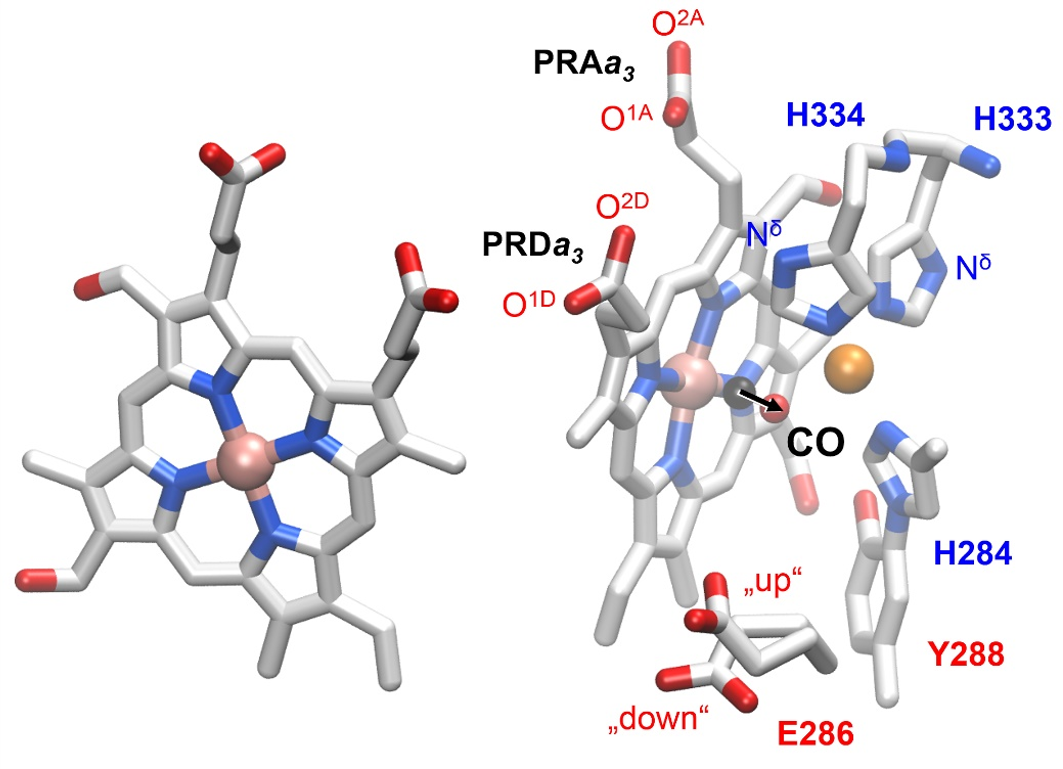


Figure S3. The active site of CcO from *R. sphaeroides* (PDB code: 2GSM) with the CO ligand from the crystal structure of CO-inhibited CcO from *B. taurus* (PDB code: 3AG1), including heme a and the binuclear center (BNC, comprised of heme a_3_ and Cu_B_). The arrow on the CO ligand at heme a_3_ indicates the direction of the $\vec{\boldsymbol{p}}$ dipole vector in our computation. The propionic acid side chains of heme a_3_ are referred to as PRAa_3_ and PRDa_3_. For E286 the “up” and “down” conformations are shown. The histidine residues coordinate the Cu_B_ site while tyrosine Y288 has catalytic relevance.

**
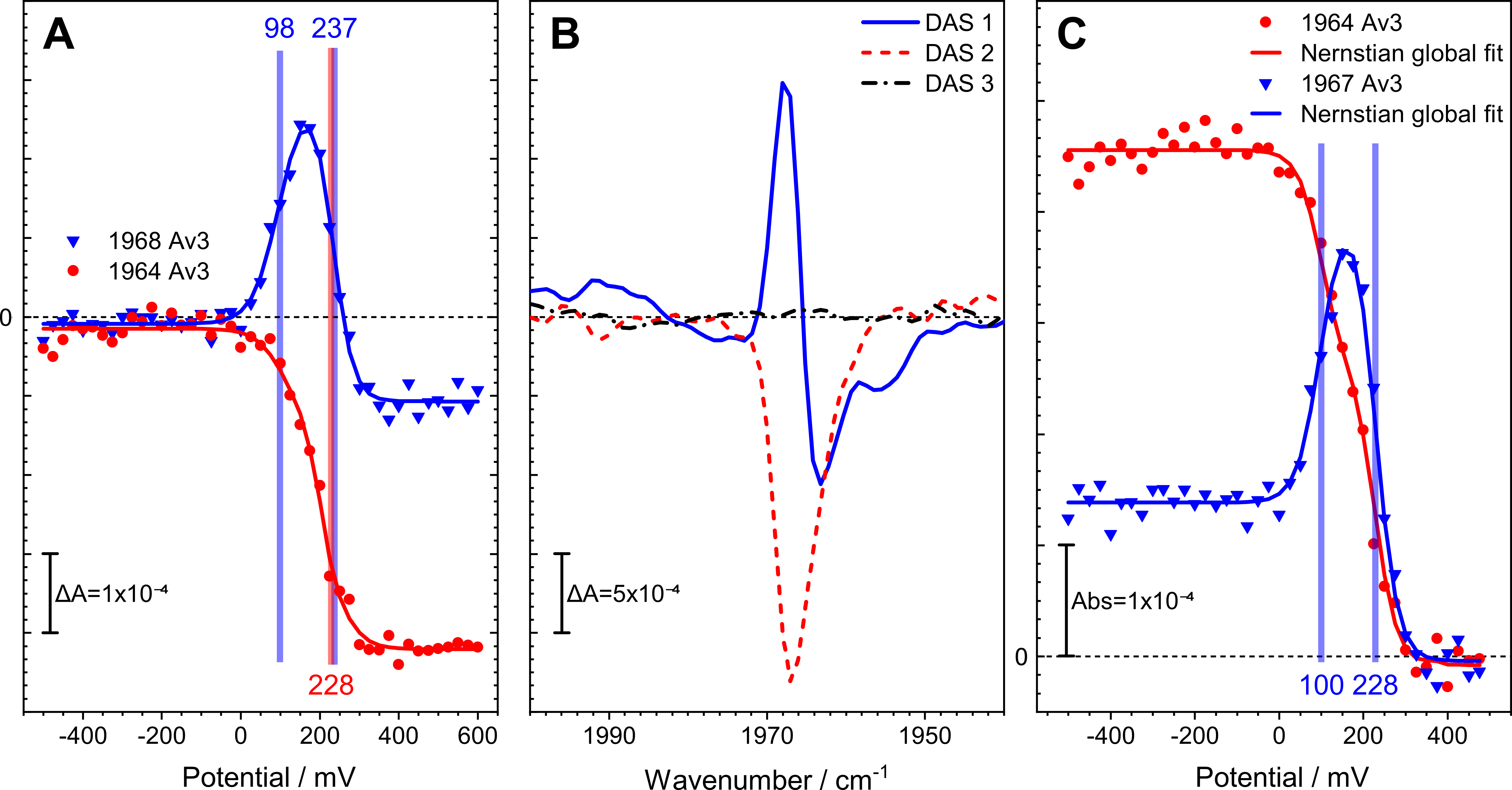
**

Figure S4. Redox titration Global Fits in the CO spectral region in H_2_O buffer. Potentials are given against Ag/AgCl. (A) Intensities of the peaks at the apparent peak positions of 1968 and 1964 cm^-1^ (same as in Figure 5), taken from the raw difference spectra with background at reducing potential (-500 mV) of Figure 3A. The scatter plots represent the average of three single wavenumbers (1967−1969 and 1963−1965 cm^-1^), centered on the peak position. Globally fitting these data with three sigmoidal functions results in the solid traces. The third component is necessary to correct for the different baseline offsets of the two species and is only relevant to our global fit program (that was originally built for cyclic reactions). The vertical lines show how the midpoint potentials of the transition at higher potentials converge even after introducing the third component (fitted values are 98±34, 228±25, and 237±27 mV, highlighting that the latter two are equal). (B) Decay-associated spectra (DAS) relative to the two redox species. These spectra represent the amplitudes of the three observable states’ Nernstian transitions being fitted to the dataset. Note that DAS 3 is only representative of the artificial species necessary for the offset compensation and carries no spectral information. (C) Intensities of the same CO peaks as in panel A, but with background at oxidizing conditions. The artificial background was obtained by averaging the three most oxidized single-channel spectra and re-calculating the absorbances. The scatter plots represent the average of three single wavenumbers, centered on the peak position (as in panel A). The Nernstian global fit (two 1-electron transitions) applied to the two species (solid lines) shows that the redox potentials associated with the transitions are 100±17 and 228±12 mV.


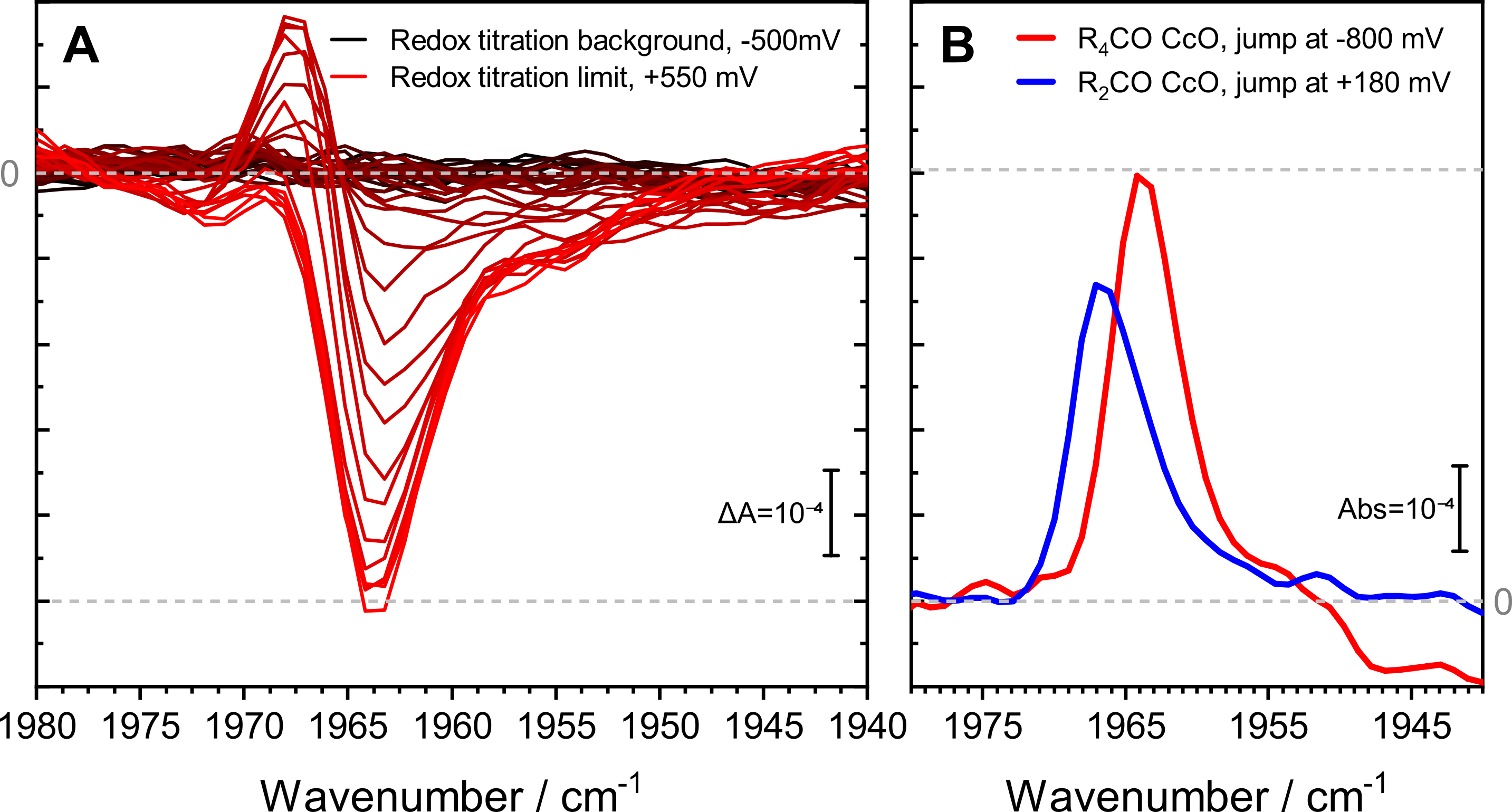


Figure S5. Comparison between the CO signature during the initial titration and in the steady state spectra obtained subsequently. The dataset refers to CcO in isotopically labelled lipids and D_2_O electrolyte buffer. (A) Overlaid lines show the raw data resulting from the initial redox titration (25 mV steps from -500 to +550 mV vs Ag/AgCl, background at -500 mV), with colors changing from black (reducing conditions) to red (oxidizing conditions). (B) Baseline-corrected data from the steady-state difference spectra (R_2_CO in blue and R_4_CO in red). The dashed grey lines illustrate that it is possible for CO to completely rebind even when changing from oxidizing to reducing conditions. The data are replotted from Figure 4.


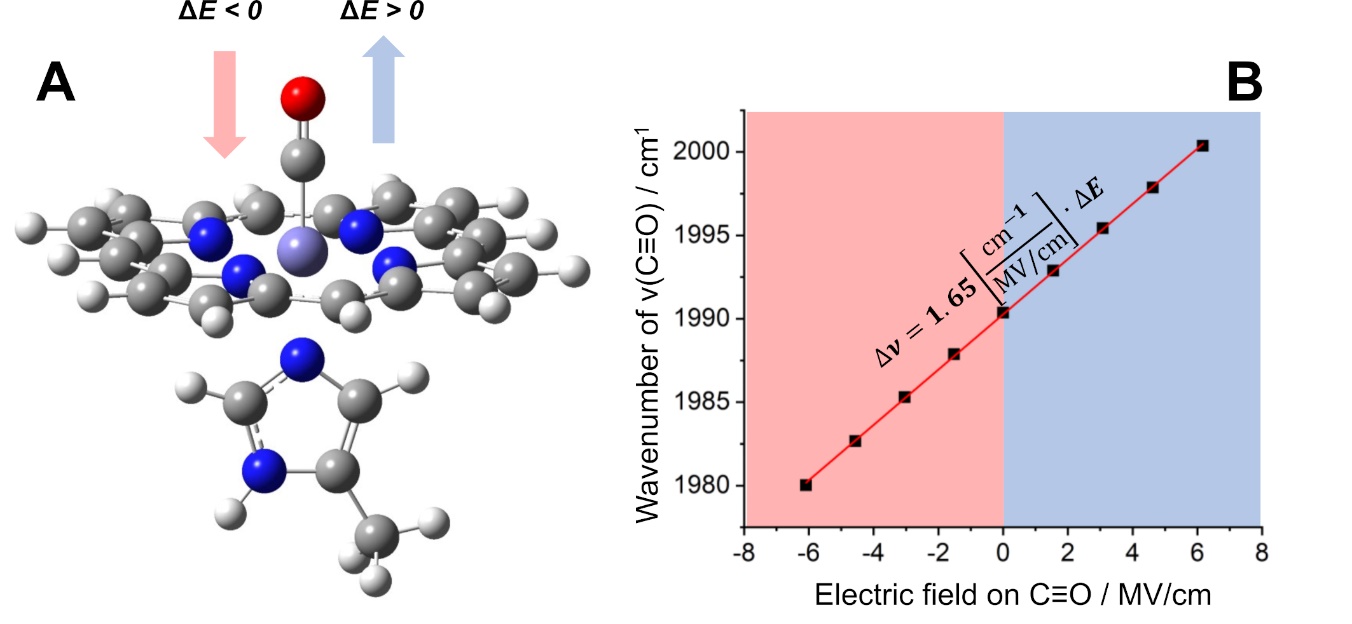


Figure S6. Density functional theory-based assessment of the direction of the vibrational Stark shift of the CO ligand. (A) Structure of Fe^2+^ porphyrin complex with axial methyl imidazole and CO ligands as minimal model system was optimized at the BP86 level of theory with TZVP and 6-31G* basis sets used for the Fe and all other atoms, respectively, using Gaussian [1]. Electric fields were applied during optimization and normal mode analysis, to obtain CO stretch frequencies, as indicated by the arrows: a negative field (*i.e.* ΔE < 0) and positive field direction (*i.e.* ΔE > 0) is indicated by the red and blue arrows, respectively. (B) Normal mode frequencies of the CO stretch (without scaling factor) are plotted against the electric field on the CO bond, as defined in the methods section of the main text. As indicated by the underlying colors (blue, red) corresponding to the arrows in panel A, a negative electric field leads to a red-shift, whereas a positive electric field causes a blue shift. This results in a Stark tuning rate of 1.65 cm^-1^/(MV/cm) at the chosen level of theory, which is in line with experimental value of $\left| \boldsymbol{\Delta}\vec{\boldsymbol{\mu}} \right|\boldsymbol{/f=}\boldsymbol{2.6}$ cm^-1^/(MV/cm) with f ≈ 2.


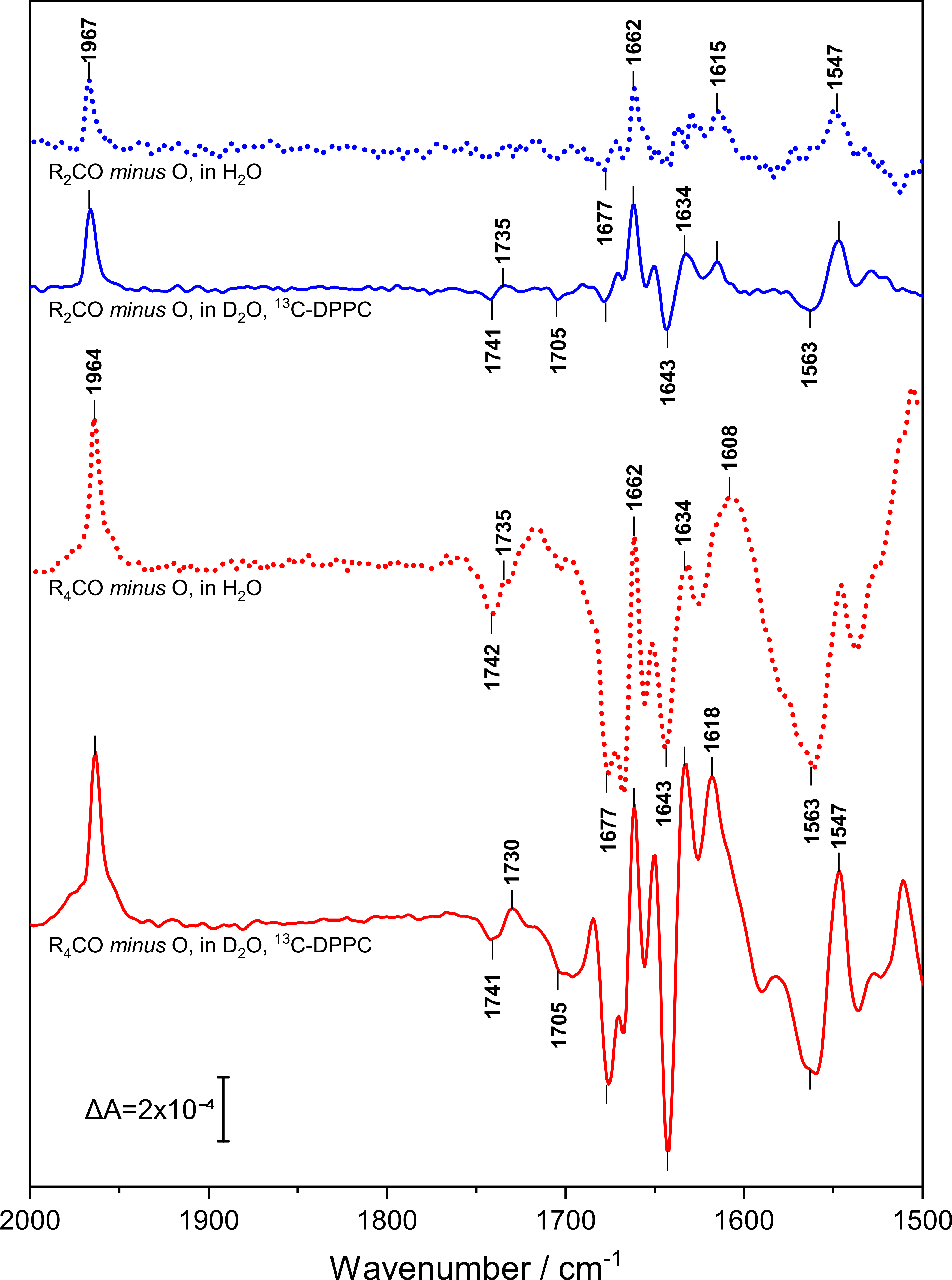


Figure S7. Comparison between steady-state difference spectra in H_2_O (dotted lines, same sample as in Figure 3 and Figure S4) and in D_2_O (solid lines, replot from Figure 4). The Potentials for the R_2_CO states were obtained by setting the potential at the maximum intensity of the 1967 cm^-1^ CO signal for both datasets. The R_2_CO and R_4_CO species in H_2_O buffer solution were accumulated at +175 and -400 mV vs Ag/AgCl, respectively. The annotated band positions are discussed in the main script. Peaks with the exact same position in H_2_O and D_2_O are omitted for clarity and only indicated by ticks. Exchanging H_2_O to D_2_O decreases noise between 1800 and 1600 cm^-1^ (from 0.12×10^-3^ to 0.05×10^-3^ rms).


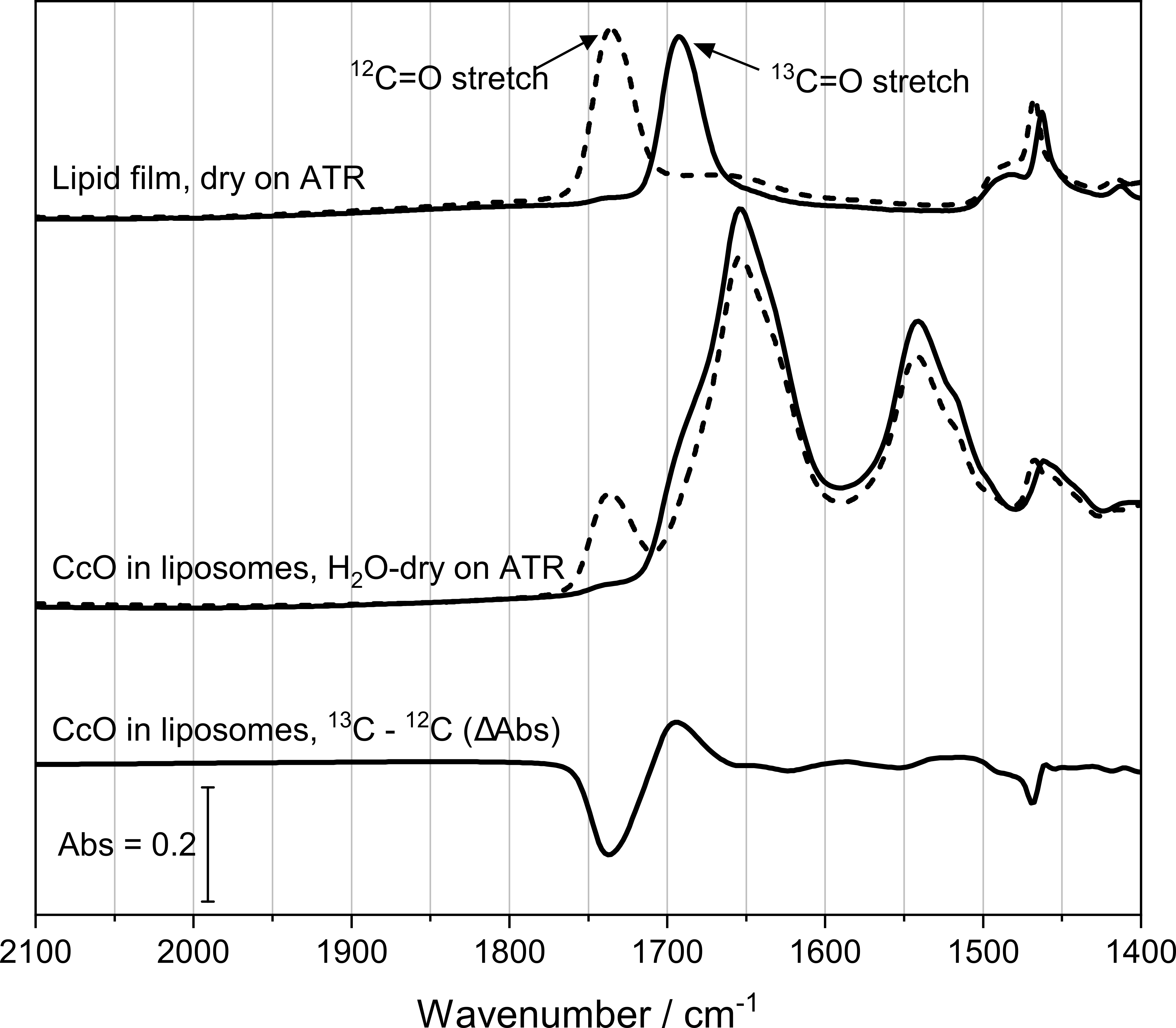


Figure S8. ATR FTIR reference spectra for the ^13^C isotopically labeled lipids. From top to bottom: absolute spectra of dry Dipalmitoylphosphatidylcholine liposomes (DPPC, dashed line) and isotopically labelled lipids (^13^C_40_-DPPC, solid line). The band at 1735 cm^-1^ or 1692 cm^-1^ has been assigned to the C=O stretching vibration of the ester bond. Below, absolute spectra of dry Cytochrome c Oxidase from *R. sphaeroides* in DPPC (dashed line) and ^13^C_40_-DPPC (solid line). The difference spectrum at the bottom was obtained by subtracting the two previous spectra. Using ^13^C_40_-DPPC shifts the broad ν(C=O) absorption band by approximately 40 cm^-1^ and liberates the area of interest, *i.e.*, 1750–1700 cm^-1^, from any possible effect belonging to DPPC.


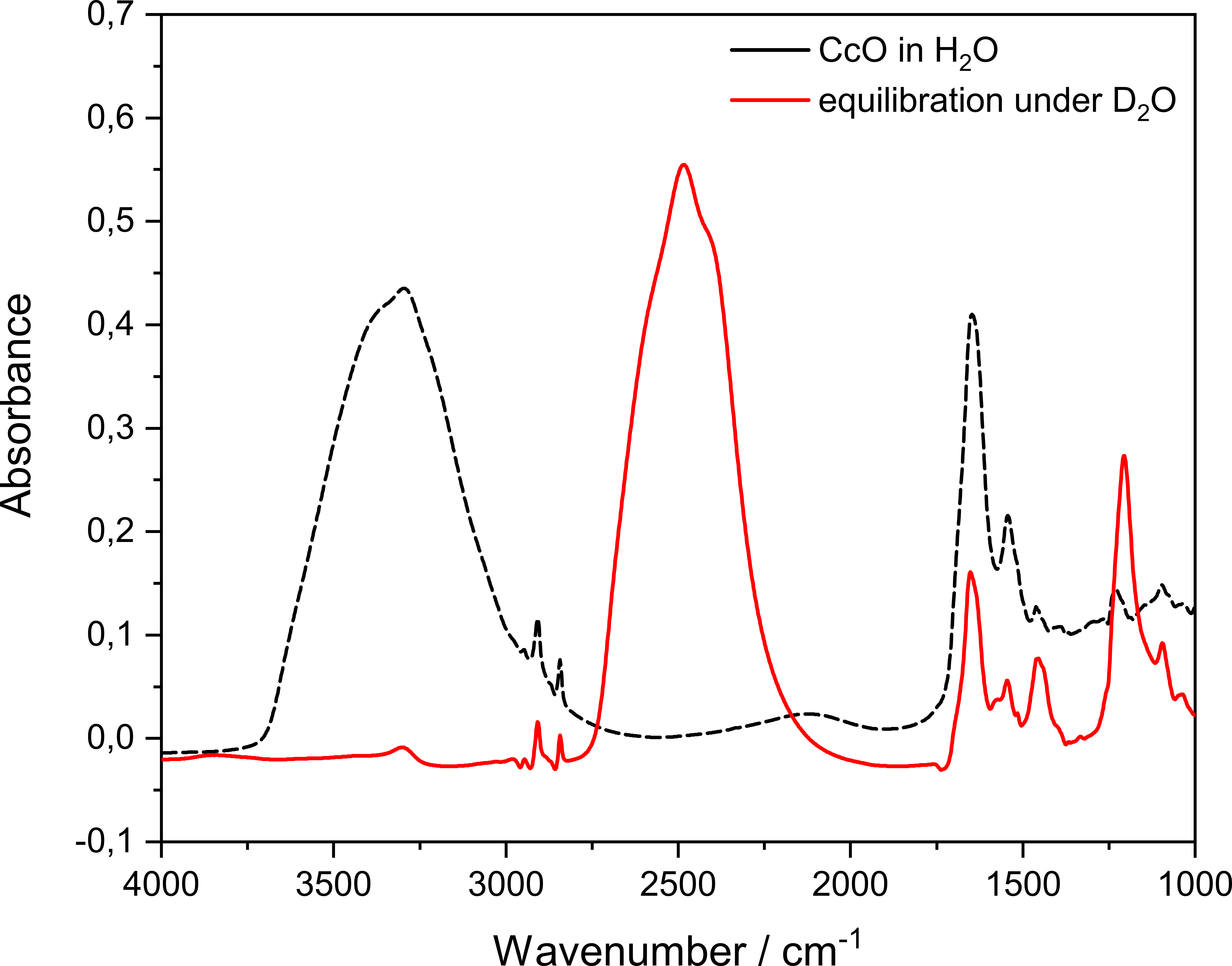


Figure S9. Preparation of the D_2_O-substituted sample of CcO in isotopically labelled lipid vesicles. The sample was originally prepared in H_2_O (black dashed line, see sample preparation in Materials and Methods), then it was slowly dried under N_2_ flow in the anaerobic chamber. The sample was then deuterated with D_2_O, re-dried and put under D_2_O electrolyte buffer. Exchanging H_2_O to D_2_O shifts the ν_2_ bending band by approximately 430 cm^-1^. The final sample shows the successful D_2_O substitution in the protein’s amide region from 1700–1400 cm^-1^ (solid red line).

References:

[1] Gaussian 16, Revision C.01, M. J. Frisch, G. W. Trucks, H. B. Schlegel, G. E. Scuseria, M. A. Robb, J. R. Cheeseman, G. Scalmani, V. Barone, G. A. Petersson, H. Nakatsuji *et al.*, Gaussian, Inc., Wallingford CT, 2016.
